# Asymmetric Engagement of Amygdala and Its Gamma Connectivity in Early Emotional Face Processing

**DOI:** 10.1371/journal.pone.0115677

**Published:** 2015-01-28

**Authors:** Tai-Ying Liu, Yong-Sheng Chen, Jen-Chuen Hsieh, Li-Fen Chen

**Affiliations:** 1 Institute of Brain Science, National Yang-Ming University, Taipei, Taiwan; 2 Integrated Brain Research Unit, Department of Medical Research, Taipei Veterans General Hospital, Taipei, Taiwan; 3 Institute of Biomedical Informatics, National Yang-Ming University, Taipei, Taiwan; 4 Department of Computer Science, National Chiao Tung University, Hsinchu, Taiwan; University College of London - Institute of Neurology, UNITED KINGDOM

## Abstract

The amygdala has been regarded as a key substrate for emotion processing. However, the engagement of the left and right amygdala during the early perceptual processing of different emotional faces remains unclear. We investigated the temporal profiles of oscillatory gamma activity in the amygdala and effective connectivity of the amygdala with the thalamus and cortical areas during implicit emotion-perceptual tasks using event-related magnetoencephalography (MEG). We found that within 100 ms after stimulus onset the right amygdala habituated to emotional faces rapidly (with duration around 20–30 ms), whereas activity in the left amygdala (with duration around 50–60 ms) sustained longer than that in the right. Our data suggest that the right amygdala could be linked to autonomic arousal generated by facial emotions and the left amygdala might be involved in decoding or evaluating expressive faces in the early perceptual emotion processing. The results of effective connectivity provide evidence that only negative emotional processing engages both cortical and subcortical pathways connected to the right amygdala, representing its evolutional significance (survival). These findings demonstrate the asymmetric engagement of bilateral amygdala in emotional face processing as well as the capability of MEG for assessing thalamo-cortico-limbic circuitry.

## Introduction

Rapid detection of facial expressions and emotional salience is critical for social communication and interaction. A dual-route model [1] has been proposed that rapid brain responses to emotional facial expressions through two pathways, one from the subcortical colliculo-pulvinar to the amygdala and the other from cortical visual areas to amygdala, within 120 ms [2]. Such rapid amygdala engagement is biologically crucial for survival under threatening confrontation [3,4].

The amygdala is a key neural substrate of emotion processing and its involvement of the left and right sides has been proposed in the literature [5–7]. A function-specific processing difference between the left and right amygdala has been noted in reviews [8–10] and amygdala-lesion studies [11,12]. Markowitsch [9] proposed that the left amygdala is more involved in language-related affective information encoding and detailed feature extraction, whereas the right amygdala is involved in image-related affective information retrieval and is engaged more in a fast/gross analysis of affect-related information than the left. The impaired skin conductance responses evoked by arousing stimuli in an amygdala-lesion study [11] supported the hypotheses of an arousal-value decoding function for the left amygdala and an autonomic-activation function for the right. However, a predominant role of the left amygdala in emotion processing has been proposed [8]. Baas et al., in a meta-analysis of 54 fMRI and PET studies, found that there were more reports of left amygdala engagement (41 out of 54 studies) than right (30 out of 54 studies). This left-predominant phenomenon may be explained by rapid habituation of the right amygdala and sustained activity of the left amygdala. Wright et al. found faster habituation of the right amygdala compared with the left amygdala and more activation of the left amygdala in response to the contrast of repeated fear and happy faces [13]. However, whether the characteristics of fast habituation in the right amygdala and sustained activity in the left amygdala exist in early emotion perception, especially within 100 ms, is still unclear. Using neuroimaging tools to determine the temporal profiles of the left and right amygdala activity would help clarify the asymmetric activity and habituation rates of the left and right amygdala in early perceptual processing of emotions.

Magnetoencephalography (MEG), a technique with high spatiotemporal resolution, is emerging as a valuable functional neuroimaging tool for exploratory investigation of brain activity and connectivity [14]. Previous studies have provided evidence to support the feasibility of using MEG to investigate emotional modulation of cortical and subcortical structures [15–18]. Two of these human studies also demonstrated the existence of a dual-route model, a quick pathway to the amygdala via subcortical thalamus regions and a slow one via visuocortical regions [1]. Garrido et al. [18] applied dynamic causal modeling to auditory oddball responses while viewing emotional faces. They reported that the subcortical pathway expedited the evaluation of salient sensory input, which was not limited in the context of fear. Luo et al. [15] reported rapid onset of gamma synchronization at the thalamus (10–20 ms) and the amygdala (20–30 ms) of subjects in response to fearful facial expression while performing a gender judgment task. They showed that the response through this subcortical pathway was faster than that through visual cortex (40–50 ms). Converging findings in functional neuroimaging studies have demonstrated that the fast subcortical route, the thalamo-amygdala pathway, can facilitate the rapid detection of faces and fear expression [19,20]. However, it is still unclear whether facial expressions other than fear, including positive and neutral faces, activate this subcortical route.

In addition to the dual-route model, a “many-road” model for the amygdala has been proposed recently [21,22]. In the review article [22], the role of the amygdala in visual processing, especially for salient, emotional, and socially charged visual stimuli, was investigated by anatomical and physiological data. They suggested that the amygdala, as well as the pulvinar, coordinates cortical functions during evaluation of the biological significance of affective visual stimuli. This new model also implies that the cortex plays a more important role in emotion processing than traditionally assumed, including the temporal, parietal, and frontal cortices. Among these cortical structures, the superior temporal sulcus (STS) has an anatomical and functional reciprocal connection with the amygdala [23]. This region, especially the posterior part, was suggested to have functional relevance to face perception for detecting changeable facial features [24] as well as to social perception for signaling the actions of another individual [25]. Furthermore, a recent MEG study [17] demonstrated that the STS can help distinguish facial expressions at approximately 45 ms after stimulus onset, indicating that facial features can be decoded or evaluated in the very early perceptual stage. Hence, it is plausible that the STS plays an essential role in the cortical-amygdala pathway.

In the present study, we addressed two questions. First, are the temporal patterns of left and right amygdala activity in response to different facial expressions distinct in early emotion perception (within 100 ms after stimulus onset)? Second, which model, dual-route or many-road, is more representative for each emotion? The two hypotheses of this study are as follows: 1) that the right amygdala would be activated earlier and habituate faster in response to threat-related faces than to positive faces, whereas the left amygdala would be activated longer in response to emotional faces than to neutral faces; and 2) that dual-route processing would be engaged only in response to threat-related faces and not in response to positive or neutral faces.

To answer these questions, the brain dynamics of neural responses to emotional face images was investigated using MEG. We focused on gamma-band oscillations of emotional responses, which act as an integrative mechanism underlying cognitive processing [26] or emotion processing [27]. A beamforming technique [28] was used to reconstruct the brain activity in a voxel-wise manner, followed by an effective connectivity analysis using Granger causality analysis (GCA) [29] to determine the directional relationship between cortical and subcortical regions. Previous fMRI studies have reported that amygdala activity highly depends on relatively passive or implicit processing of an emotion [2]. The reduction of amygdala responses to emotional facial expressions, when the demand for explicit emotion recognition is increased, is a common observation across studies [19,30–32]. A meta-analysis of 385 PET and fMRI studies concluded that passive/implicit processing of emotions is associated with a higher probability of amygdala activation than active task instructions [33]. Based on these findings, we adopted a gender judgment task on visually displayed face images to engage less attentional loading of emotional stimuli to investigate amygdala activity. Finally, we determined connectivity models and compared gamma activity of the left amygdala with that of the right amygdala for each facial expression.

## Materials and Methods

### Subjects

Twenty-four healthy volunteers (nine male, mean age 36.6 ± 11.3 yrs) were enrolled. All participants were right-handed as assessed by the Edinburgh Handedness Inventory and had normal or corrected-to-normal vision. They underwent a Mini International Neuropsychiatric Interview by a psychiatrist in Taipei Veterans General Hospital before the experiments to exclude possible morbidity associated with psychiatric illness. All subjects signed written consent forms and were financially compensated for their participation. The study was approved by the Institutional Review Board at Taipei Veterans General Hospital.

### Stimuli and experimental design

Face images with expressive emotions including neutral, sad, happy, and angry, were displayed in a random order at the center of a back-projected translucent screen, located 100 cm in front of the subject, and subtended 14° (width) by 17° (height) of visual angle. To avoid ethnic/cultural differences in emotion processing, the facial expression images used in the present study were all collected from Taiwanese and processed into gray-scaled, face-only images. Each face stimulus was presented for 1500 ms, followed by a preparatory blank image (jittered with a mean duration of 1000 ms) and a response cue of 500 ms, using STIM2 software (Neuroscan Inc.). There were 72 trials for each emotion. Subjects were instructed to perform a gender discrimination task on each presented face image by lifting their left or right index finger for male or female, respectively, when a response cue was displayed. A training phase was conducted before the MEG recordings.

### MEG and MRI recordings

Event-related MEG signals at a sampling rate of 1000 Hz with a 0.03–330 Hz band-pass filter were recorded using a whole-head 306-channel neuromagnetometer (Vectorview, Elekta-Neuromag, Helsinki, Finland). Trials contaminated by eye movements or containing deflections exceeding 9000 fT/cm were discarded. MEG signals were processed using the signal space projection method [34] to remove urban interference and obtain noise-free trials for source analysis. Three fiducial landmarks (nasion and bilateral preauricular points) and four head position indicator coils were localized using the Isotrak system (Polhemus Navigation Sciences, Colchester, Vermont, USA). The fiducial points allowed precise co-registration of the MEG and structural magnetic resonance imaging (MRI) data. The anatomical MRI data were acquired by a GE Signa EXCITE 1.5 T system using an 8-channel phased-array head coil with a high-resolution, T1-weighted, 3D fast spoiled gradient-recalled echo sequence (3D FSPGR, TR = 8.67 ms, TE = 1.86 ms, inversion time = 400 ms, matrix size = 256 × 256 × 124, and voxel size = 1.02 × 1.02 × 1.5 mm^3^). Some of the present data have been published previously [27].

### MEG source analysis

Beamformer-based analyses using the trial-by-trial data were performed for each emotion. The noise-free MEG data were filtered at a frequency band of 35 to 55 Hz (gamma rhythm). These gamma-band signals were then analyzed through a beamforming method [28], which yielded a spatial filter that was designed, under a unit-gain constraint, for each targeted brain location to minimize the variance of filtered activities over a time interval of interest. We in the present study used a window of 300 ms (from 0 ms to 300 ms after stimulus onset) as time interval of interest, which is around 10 cycles of gamma activity, to estimate the spatial filter for accurate source reconstruction. A homogeneous spherical conductor model was used in the calculation of forward solution. Tikhonov regularization (Tikhonov 1977) was adopted as a smoothness term, where the parameter of regularization indicates the noise suppression factor. When noise level is high, more regularization has to be applied to gain more stability of the solution, even though more spatial resolution is sacrificed.

For each location in the brain, a sliding window of 30 ms with a shift of 5 ms was applied [35,36] to calculate the temporal dynamics of power ratio of brain activity reconstructed by using the estimated spatial filter. The window was sliding with its center shifting from 35 ms to 165 ms. Within the sliding window, the beamformer estimated the gamma band activation index (GBAI), which was a pseudo F-statistic value, by calculating the power ratio of the reconstructed gamma activity between the active and control states. Here the interval of control state was chosen from 300 ms to 200 ms before stimulus onset. Two simulation studies (S1 Fig. and S2 Fig.) were conducted to further demonstrate the effectiveness of the proposed method for estimating temporal power dynamics of gamma activity. The whole-brain GBAI map was then obtained by iteratively scanning through the brain volume using the same procedure with an isotropic spatial resolution of 4 mm. Because angry and happy emotions have relatively high arousal and sad emotion has relatively low arousal [30,33,37], only the data of angry and happy emotion conditions were analyzed in this study because of their similar arousal levels.

### Localization and extraction of cortical gamma activity

**Figure 1:**
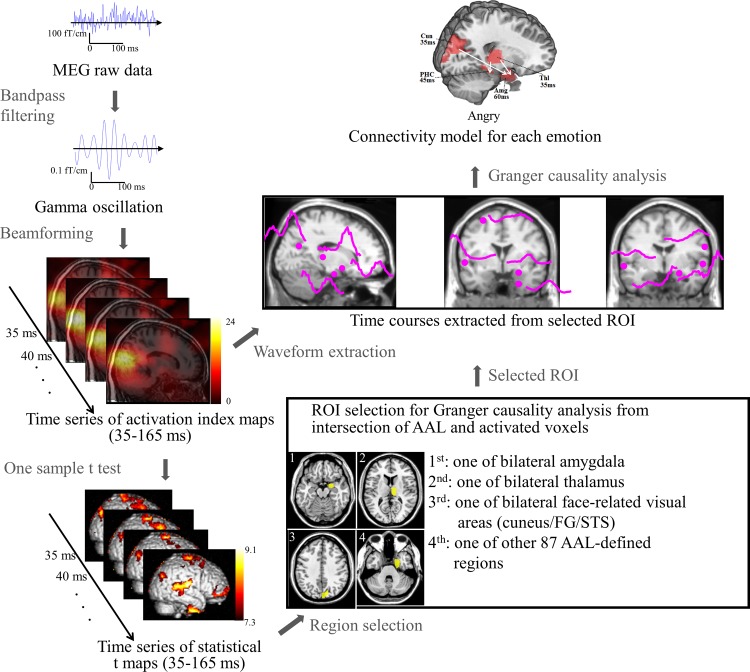
Schematic flow of processing the event-related gamma activity. The analysis was employed to estimate spatiotemporal profiles of amygdala activity, to identify regions of interest (ROI) based on the intersection between survived voxels from one sample t-test and automatic-anatomical-labeling template, and to perform effective connectivity analysis of angry, happy and neutral emotions.

The overall processing flow of the analysis and integration of functional and structural imaging data is summarized in Fig. 1. First, for each individual the deformation field was determined by aligning individual T1-weighted MRIs to a standard stereotactic space (Montreal Neurological Institute, MNI, space) using BIRT software [38]. For group analysis, the corresponding GBAI maps of each individual obtained from the beamformer were then aligned to the same standard stereotactic space by applying the resolved deformation field with an isotropic voxel size of 2 mm.

The whole-brain group analysis was used to locate the brain regions with significant activity in the whole brain and then to calculate the mean temporal profile of gamma activity within the located brain regions for further connectivity analysis. In order to provide a unified approach of model selection in GCA for all emotions, the activation foci were determined by combining both functional and anatomical information to accommodate inter-subject variability in functional neuroanatomy. To obtain functional activation map, a one-sample t-test was first conducted on GBAI maps at each time point using SPM2 software (http://www.fil.ion.ucl.ac.uk/spm). The significance level was set at uncorrected p < 10^−7^ (t(23) = 7.3) and cluster extension > 1000. This threshold was also met by the false discovery rate (FDR) corrected p value of < 10^−5^. Compared to an FDR corrected p-value, an uncorrected p-value, corresponding to a specific t-value for all time points and conditions, is less dependent on the distribution of the underlying signal. The uncorrected p value (t(23) = 7.3, p < 10^−7^) remained significant after multiple testing correction across time (met Bonferroni corrected p < 10^−5^). The union of the significant voxels survived from all time points was then determined as the functional activation map. As to anatomical information, we employed the automated anatomical labeling (AAL) atlas [39] to parcel the cerebral cortex into 90 brain regions. Finally, the intersection area between each AAL-defined region and the functional map was identified as a Region-of-Interest (ROI) if it was larger than 10 voxels. The time course of the mean gamma activity within each ROI was calculated for connectivity analysis.

To further compare the activity between the left and right amygdala, the mean gamma activity of the left and right amygdala ROIs determined by the above-mentioned procedure was analyzed by a 2-tailed paired t-test at each time point. An effect size was computed by a standardized measure (Cohen’s d) [40].

### ROI identification and GCA

We performed effective connectivity analysis using GCA [29] to investigate directional interaction among brain regions. The GCA method has been widely used in neuroimaging research [11,41,42], which is a statistical procedure based on lagged time-series regression models to determine the ability of one time-varying signal to predict the future behavior of another.

The GCA in this study was performed by using the time series of GBAI in the above-defined ROIs with the Casual Connectivity Analysis toolbox [43] in Matlab. For each subject, the time series of GBAI were calculated by averaging across significant voxels in the ROIs at each time point in each facial expression. To ensure that the mean, variance, and auto-covariance of a series remained constant over time, a Dickey-Fuller test (*p* < 0.01) was performed to verify whether the time series of GBAI were covariance-stationary. In the present study, a model of order two was selected to identify the main characteristics of the networks. Kopell et al. [44] showed that gamma oscillations support robust lag synchronization between two sites of up to 8–10 ms by simulating physiological parameter regimes. According to synchronous firing within a ±10 ms time lag [45] and neuronal synchronization approximately 10 ms [44,46], a time lag of 10 ms was chosen [41].

To examine whether core cortical regions of face perception (including cuneus, fusiform gyrus (FG), and STS) and subcortical region (thalamus) are involved in the efferent pathway to the amygdala during processing of emotional faces, an effective connectivity network consisting of four regions was constructed using the lagged multivariate vector auto-regressions on the time series of GBAI. For model space specification, four out of 90 AAL-defined cerebral regions were selected as follows. The first one was either the left or right amygdala because of the essential role of amygdala in the dual-route model. The second and third regions were selected from the bilateral thalamus and from the three bilateral face-related cortical regions (cuneus/FG/STS), respectively, to ensure that both thalamus-amygdala and cortical area-amygdala pathways were evaluated concurrently in the connectivity model. The fourth region was then selected from the remaining 87 AAL-defined cerebral regions. In total, there were 2,088 (2 × 2 × 6 × 87) models to be examined for each emotion.

For each model under examination, the significance level of the directed link between each pair of regions was first estimated by GCA [41,47] for each subject (F-test, p < 0.05, uncorrected) and then verified by group analysis. Significance of connections at the group level was examined by using a binomial test (p < 0.0321, 17/24 subjects; that is, at least 17 of the 24 subjects passed the F-test at the individual level) [42,48]. For a success probability of 0.5 (a connection exists or not) in a binomial distribution with 24 trials, the minimal number of successes is 17 so that the critical number of 17 or more successes (the number of subjects that had a connection) was selected. Finally, the models containing two links to amygdala, one from thalamus and the other from face-related areas, were determined as the representative models for each emotion.

## Results

For each emotion, the spatiotemporal GBAI maps obtained from the beamforming method with a 5-ms sliding window illustrate the significant gamma-band activity of the whole brain (see S3 Fig.).

### Temporal profiles of each emotion in the amygdala

**Figure 2:**
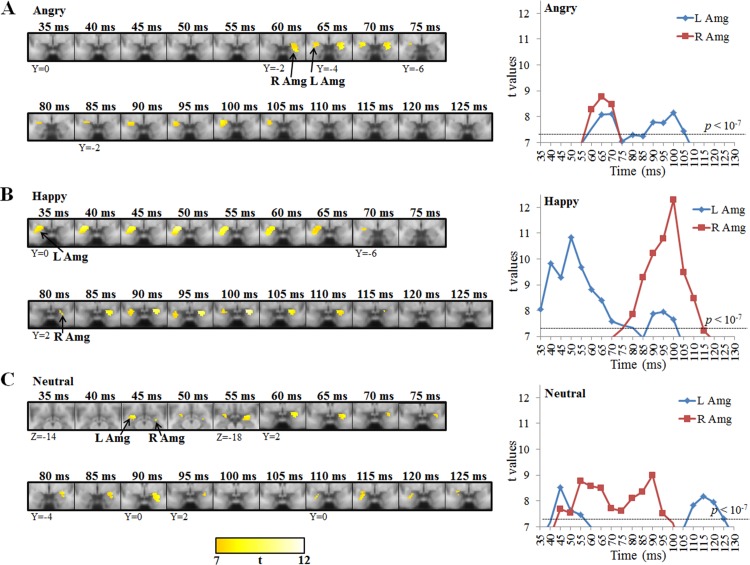
Spatiotemporal profiles of the left and right amygdala activity for each emotion. The left panel showed the activated regions in response to (A) angry, (B) happy, and (C) neutral faces (extended cluster ≥ 10 voxels and t-value ≥ 7). The temporal curves in the right panels are plotted from the peak voxels. Dotted lines denote the significant level of uncorrected p < 10^−7^. *Abbreviations*: L, left; R, right; Amg, amygdala.

The spatial patterns and temporal profiles (35–125 ms) of the bilateral amygdala activity (t-value ≥ 7, extended cluster size ≥ 10 voxels) are illustrated in the left and right panels of Fig. 2, respectively. We found that angry faces elicited amygdala activity on the right side at 60–70 ms and on the left side at 65–70 ms and 90–105 ms (Fig. 2A). In response to happy faces, the left amygdala was activated at 35–70 ms and 90–100 ms, and the right amygdala was activated at 80–110 ms (Fig. 2B). In response to neutral facial expressions, right amygdala activity was detected at 45–95 ms and left amygdala activity at 45–55 ms and 110–125 ms (Fig. 2C). The angry (85, 90, and 95 ms; left > right) and happy facial expressions (95 and 100 ms; right > left) showed significant differences of gamma-band activity between the left and right amygdala (paired t(23) ≥ 2.61, p < 0.016, effect size ≥ 0.53). Neutral facial expressions did not show significantly different activation. The finding of distinct temporal profiles of the left and right amygdala activity for different emotions confirmed our first hypothesis.

### Onset and peak latencies for different emotions in the amygdala, thalamus and face-related cortical regions

Table 1 summarizes the activity onset, peak latency, and corresponding MNI coordinates of the bilateral amygdala, thalamus, cuneus, FG, and STS for all expressions. The activity onset denoted the time of the significantly increased gamma oscillatory activity (t(23) = 7.3, uncorrected p < 10^−7^, met FDR corrected p < 10^−5^, effect size = 1.49), which was compared to the baseline period (from 300 ms to 200 ms before stimulus onset). The peak latency represented the time point of the maximal value of gamma activity compared to the control period.

**Table 1:** The onset and peak time points and corresponding MNI coordinates for each facial expression.

**Region**	**Angry**				**Happy**				**Neutral**			
	**Time (ms)**		**Coordinates (mm)**		**Time (ms)**		**Coordinates (mm)**		**Time (ms)**		**Coordinates (mm)**	
	**Onset**	**Peak**	**x,y,z**	**t-value**	**Onset**	**Peak**	**x,y,z**	**t-value**	**Onset**	**Peak**	**x,y,z**	**t-value**
L Amg	65	100	−29, −4, −14	8.15	35	50	−24, −2, −14	10.84	45	45	−16, 0, −14	8.51
R Amg	60	65	30, −2, −26	8.76	80	100	22, −4, −12	12.30	45	90	28, 4, −18	8.99
L Thl	55	65	−6, −22, 10	10.12	45	50	−6, −28, 8	9.82	35	85	−8, −18, −2	11.00
R Thl	35	65	2, −12, 4	10.64	35	95	18, −10, −2	10.78	35	95	12, −16, 16	11.91
L STS	35	50	−64, −46, 20	10.36	35	165	−48, −32, 10	10.25	35	80	−64, −32, 14	14.89
R STS	35	105	64, −29, 18	10.06	35	55	48, −28, 12	10.96	45	110	44, −26, −4	9.11
L Cun	35	165	−4,−72,30	9.02	35	165	−16,−56,28	8.46	40	55	−18,−58,18	8.15
R Cun	35	40	8,−78,40	8.77	35	40	6,−70,26	8.91	40	45	10,−86,40	8.60
L FG	35	75	−38,−48,−10	11.74	35	155	−26,−46,−18	11.37	35	90	−22,−2,−42	11.00
R FG	45	75	40,−6,−32	9.75	45	75	40,−44,−16	11.01	35	50	36,−48,−18	11.45

All t-values > 7.3, uncorrected p < 10^−7^; L: left; R: right; Amg: amygdala; Thl: Thalamus; STS: superior temporal sulcus; Cun: cuneus; FG: fusiform gyrus.

In response to angry facial expressions, the early event-related gamma activity in the right thalamus, bilateral cuneus, left FG, and bilateral STS was detected at 35 ms and followed by activity in the right FG (45 ms), left thalamus (55 ms), and amygdala (right, 60 ms; left, 65 ms). The gamma-band responses in the right cuneus, left STS, bilateral thalamus, right amygdala, and bilateral FG peaked at 40–75 ms, which was earlier than the right STS and left amygdala at 100–105 ms and than the left cuneus at 165 ms.

Happy faces elicited early gamma activity in the bilateral STS, bilateral cuneus, left FG, left amygdala, and right thalamus at 35 ms, which was followed by that in the left thalamus (45 ms), right FG (45 ms), and right amygdala (80 ms). With regard to the time points with peak t-values, the right cuneus, the left amygdala, left thalamus, and right STS responded at 40–55 ms, earlier than the right FG (75 ms), right thalamus (95 ms), right amygdala (100 ms), left FG (155 ms), left cuneus (165 ms), and left STS (165 ms). Notably, the onset and peak latencies of the left amygdala were earlier than those of the right.

In response to neutral faces, the bilateral thalamus, bilateral FG, and the left STS were activated at 35 ms, followed by the bilateral cuneus (40 ms), bilateral amygdala (45 ms), and right STS (45 ms). As to the time points with peak t-values, all of the amygdala, thalamus, FG, and STS were activated approximately 80–110 ms, except the left amygdala (45 ms), bilateral cuneus (45–55 ms), and right FG (50 ms).

### Effective connectivity from subcortical and cortical regions to the amygdala

**Figure 3:**
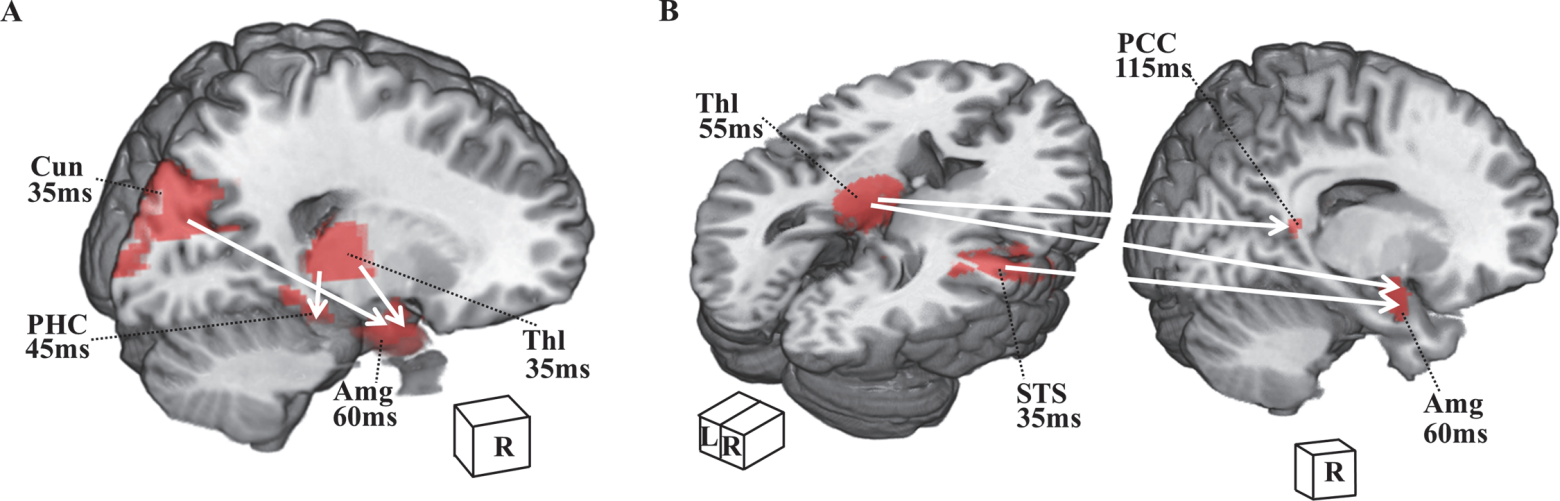
Cortical and subcortical routes to the amygdala for early perceptual processing of angry emotion. White arrows indicate significant connections during 35–165 ms directional influences between regions in red for angry faces in (A) and (B). *Abbreviations*: L, left; R, right; Amg, amygdala; Thl, thalamus; Cun, cuneus; PHC, parahippocampal cortex; SFC, superior frontal cortex; STS, superior temporal sulcus; PCC, posterior cingulum.

A GCA was performed for the duration of 35–165 ms with a four-region model for each emotion category. The significant effective connectivity (p < 0.0321, effect size ≥ 4.90) between the regions is displayed in Fig. 3.

Two connectivity models in processing of angry emotion were found, as shown in Fig. 3. The first model consisted of pathways from the right thalamus and from the right cuneus to the right amygdala (Fig. 3A). The second model consisted of pathways from the left thalamus and from the right STS to the right amygdala (Fig. 3B). These two models also showed that the right thalamus affected the activation of the right parahippocampal cortex (Fig. 3A) and the left thalamus influenced the activation of the right posterior cingulate cortex (Fig. 3B). With respect to happy and neutral facial expressions, no dual-route model was found. These data provided evidences that dual-route processing of the right amygdala from both subcortical (bilateral thalamus) and cortical (cuneus and STS) regions existed only in negative (angry) face perception, but not in positive (happy) or neutral face perception.

## Discussion

Our results show asymmetric engagement of the left and right amygdala in early perceptual processing of emotional faces. The right amygdala responded to angry faces (60–70 ms) earlier and shorter than to happy faces (80–110 ms); whereas the left amygdala responded to happy faces (35 ms) earlier than to angry faces (65 ms) (Fig. 2). With GCA, we found the evidence of the dual-route model, thalamo-amygdala and visuocortical-amygdala pathways, in angry face perception. No evidence was found in happy and neutral face perception. These data are in keeping with the view that a dual route facilitates processing of threatening information in humans.

### Asymmetric activation of amygdala in early perceptual processing of emotional faces

We found that the left amygdala activity lasted longer than the right in response to emotional faces, but not to neutral ones. Moreover, our data showed that the left amygdala responded to happy faces (35 ms) earlier than to angry ones (65 ms). These results implicate the left-lateralized involvement of decoding or evaluating expressive stimuli in the early perceptual processing of emotion. It has been proposed that happy facial expression involves greater physical changes of the facial features, including the mouth, compared to negative facial expressions [49]. Our data are also in line with a previous report [8] suggesting a predominant role of the left amygdala in emotion perception. The findings in the present study provide evidence that there could be a left-lateralized amygdala preference in decoding expressive information during the very early perceptual period.

Relative to the left side, the right amygdala is more responsible for autonomic arousal. Our results showed that the right amygdala was engaged in processing angry emotion more quickly (60–70 ms) than processing happy emotion (80–115 ms). A previous patient study demonstrated that right amygdala damage resulted in the deficit of an autonomic response as measured by skin conductance [11]. Williams et al. [50] reported that the increased amygdala responses to negative facial stimuli appeared to be associated with concomitant autonomic arousal. This suggests that the function of the right amygdala could be linked to autonomic arousal: the more threatening the confrontation, the faster the processing.

Moreover, we found the right amygdala habituated rapidly for both angry and happy faces. The rapid habituation of the right amygdala may reflect efficient detection of emotional information for triggering autonomic responses. This finding of fast habituation of the right amygdala is in line with one previous fMRI study [13]. Wright et al. reported greater habituation of the right amygdala compared to the left in response to repeatedly presented emotional stimuli, suggesting the right amygdala is a part of a dynamic emotional stimulus detection system. In sum, our data might offer a more general perspective on the left and right amygdala subserving functions of detecting and delivering emotional signals, respectively.

### Salient detection of neutral faces by the amygdala

Notably, we found the bilateral amygdala was activated by neutral faces, and the right amygdala activation lasted longer than the left. In almost all previous neuroimaging studies, neutral faces were treated as control stimuli to be compared with emotional faces. Our data demonstrated that the temporal profile of the amygdala activity evoked by neutral faces was quite different from those for angry and happy faces (Fig. 2C). The right amygdala is involved in social perception. Social concepts elicit more right amygdala responses [51], and an impaired right amygdala may confer more derangement of social cognition compared to an impaired left amygdala [52]. Our data also showed that the right amygdala responses to neutral faces lasted longer than those to emotional faces, which could be due to computation of facial expressions. Neutral face computation is more demanding on the brain because these faces have no obvious features of emotional meaning. This finding supports the notion that the right amygdala could be involved in the processing of social cues prompted by faces, a specific aspect of the more general role of the amygdala in the detection of salience [53] and relevance [54]. Our results also suggest a need to reconsider the justification of using a neutral face as a control in the comparison of functional studies in the future.

### A dual route to amygdala in response to angry faces

To our knowledge, this is the first report of a dual-route model for angry face processing. Our data provide evidence of the LeDoux’s dual-route model in humans, that is, thalamo-amygdala and visuocortical-amygdala routes, while perceiving angry faces. This dual-route model has been previously proposed in both rat [1] and human studies [15,19]. These studies proposed a subcortical route that is capable of rapidly sending information to the amygdala raised by fearful stimuli. In our study, angry faces were adopted as negative stimuli, which may contain potential threatening features, similar to fearful stimuli [3,4]. Our findings of a dual route in processing angry faces suggest the generality of high-arousal negative facial expressions (e.g., angry and fearful), which could involve more effective and efficient brain processing to provide a survival advantage in detecting danger.

Another possible explanation of the dual-route model observed only in angry face perception is that the thalamus is involved in the early processing stage of negative faces. Our results showed that the thalamus was activated at similar onset times (all approximately 35 ms) but with different peak latencies in response to angry, happy, and neutral faces. Moreover, the right thalamus was engaged to process angry faces (at 65 ms) more quickly than to process happy and neutral faces (approximately 95 ms). These data suggest that the thalamus participates in processing emotional signals of faces at the very early perceptual stage. These findings are in line with a recent study using single-cell recordings [55], which demonstrated fast traces to the pulvinar (a part of the thalamus) neurons elicited by visual stimuli in monkeys. They reported that some visual-responsive neurons in the thalamus were triggered by angry faces but not by happy or neutral faces, indicating the capability of the thalamus to differentiate distinct emotional faces. Our findings suggest that when perceiving angry facial expression, the thalamus could efficiently convey emotional information to the amygdala and hence could facilitate a rapid response to potentially dangerous events.

Our data demonstrate that the rapid activation of cortical areas could affect amygdala activation through visuocortical-amygdala and STS-amygdala routes in very early emotional perception (starting at approximately 35 ms post-onset). The STS plays an important role in cortical connectivity to the amygdala. The STS is involved in detection of changeable feature of facial expressions (mouth, eyes, and eyebrows) [24] and has a strong connection with the amygdala in monkeys [23,56]. For humans, an interconnection between the STS and amygdala has been proposed as a network model for social perception, in which the STS and amygdala cooperate in processing significant social stimuli [57,58]. Moreover, the results of the present study demonstrated that the cortical responses in the occipital-temporal, parietal, and frontal cortices occur within 50 ms (Fig. S3), consistent with the findings of previous studies [17,59,60]. Our findings provide evidence of rapid cortical activity during early emotional perception, consisting of cortical and subcortical routes to amygdala for angry emotion.

### Importance and consideration of our methods

The framework of hemispheric functional specialization has been well investigated, indicating that the right hemisphere is relatively biased towards the processing of more global, holistic aspects of a stimulus, whereas the left hemisphere is relatively biased towards the processing of local, finer details of a stimulus [61]. Based on this, we speculate that binding of finer features, such as the valence/expressive features of a face, may involve nearby neural assemblies in the left hemisphere; whereas the global aspect of a face, such as arousal information, may be conceptualized by large-scale integration across distant brain regions in the right hemisphere. Our findings of more connectivity projected to the right amygdala suggest the role of the right amygdala in processing overall arousal information of faces.

To our knowledge, this is the first study to demonstrate a dual route of effective connectivity projected to the amygdala during early perceptual processing of emotions. This dual route to the right amygdala was found only in response to angry faces, which provides novel evidence of a neural mechanism underlying effective and efficient brain processing to provide a survival advantage in detecting danger in humans. This dual pathway could imply more processing of the expressive features of facing angry faces compared to happy faces, even though the subjects were instructed to judge the gender, not expression, of the face. Previous neuroimaging studies reported impaired emotional processing in patients with affective disorders [27,31,32,62]. However, whether the impairment occurs at the perceptual level or at the cognitive level in the thalamo-cortico-limbic regions remains unclear. The findings presented in this study suggest that MEG could be a potential tool to examine the alternation of thalamo-cortico-limbic circuitry engaged in early perceptual processing of emotional information for patients with affective disorders.

GCA identifies a directional influence that one neuronal population exerts on another. A general limitation of GCA connectivity analysis resides in its selection of ROIs, which has failure to consider the probability that other inputs from unselected regions (e.g., cerebellar areas) exert influences on the selected regions [42,63]. Consideration of other possible models without amygdala in future studies would enrich the understanding of neural mechanisms underlying early perceptual processing of emotional faces.

Numerous human studies have indicated that intra-regional oscillatory activity and inter-regional phase synchrony over different frequency bands are crucial as mechanisms for local-scale (∼1 cm through monosynaptic connections) and large-scale (>1 cm over polysynaptic pathways) integration of incoming and endogenous activity [64]. Among the different frequencies, rhythmic synchronization of neural discharges in the gamma band (approximately 40 Hz) may provide the necessary spatial and temporal links that bind the processing functions in different brain areas to build a coherent percept [65], for instance, perceptual binding of spatially separated static visual features in the infant brain [65]. A patient study that directly recorded amygdala gamma activity suggested that the amygdala participates in binding perceptual representations of the stimulus with memory, emotional response, and modulation of ongoing cognition, on the basis of the emotional significance of the stimulus [66]. The present study demonstrates the feasibility of using MEG to investigate local-scale (e.g., amygdala) and large-scale (e.g., thalamo-cortical and cortico-cortical) networks.

## Conclusions

This study provides the first evidence of functional asymmetry of amygdala in the early perceptual processing of emotions using GCA on MEG data. We suggest that the left amygdala could be more associated with decoding stimuli for all emotions whereas the right amygdala could be linked to autonomic arousal and the processing of social information. Our data demonstrated neural evidences for dual-route model in human, suggesting that processing of negative emotional information engages cortical and subcortical pathways connected to the amygdala. Negative affect engages subcortical pathway (thalamus-amygdala), representing its evolutional significance (survival).

## Supporting Information

S1 Fig Experiments of Simulation Study 1. This figure illustrates (A) the temporal profile of the Dipole Source 1, (B) its corresponding simulated MEG sensor signals, (C) the temporal dynamics and (D) tomographic maps of the pseudo F-statistic values calculated by using the proposed method with different sizes of sliding window (30 ms, 60 ms, 90 ms). The simulated MEG data were originated from background activity and one dipole source located at the right amygdala (x = 30, y = −2, z = −26mm, MNI coordinates) with temporal profile of gamma-band sinusoidal waves added by random noises. The structural MRI data and MEG sensor configuration here were adopted from one subject in our facial processing experiment.
(TIF)

S2 Fig Experiments of Simulation Study 2. This figure illustrates (A) the temporal profile of the Dipole Source 2, (B) its corresponding simulated MEG sensor signals, (C) the temporal dynamics and (D) tomographic maps of the pseudo F-statistic values calculated by using the proposed method with different sizes of sliding window (30 ms, 60 ms, 90 ms). The simulated MEG data were originated from background activity and one dipole source located at the right amygdala (x = 30, y = −2, z = −26mm, MNI coordinates) with temporal profile of gamma-band sinusoidal waves added by random noises. The structural MRI data and MEG sensor configuration here were adopted from one subject in our facial processing experiment.
(TIF)

S3 Fig Spatiotemporal gamma-band activity of the whole brain in response to the angry (A), happy (B), and neutral (C) faces during 35–125 ms after stimulus onset (extended cluster ≥ 10 voxels and t-value ≥ 7.3).
(PDF)
