# Supplementary figures and images for: Asymmetric Engagement of Amygdala and Its Gamma Connectivity in Early Emotional Face Processing
Source: PLoS One. 2015 Jan 28;10(1):e0115677. doi: 10.1371/journal.pone.0115677 (PMC4309641; doi:10.1371/journal.pone.0115677)

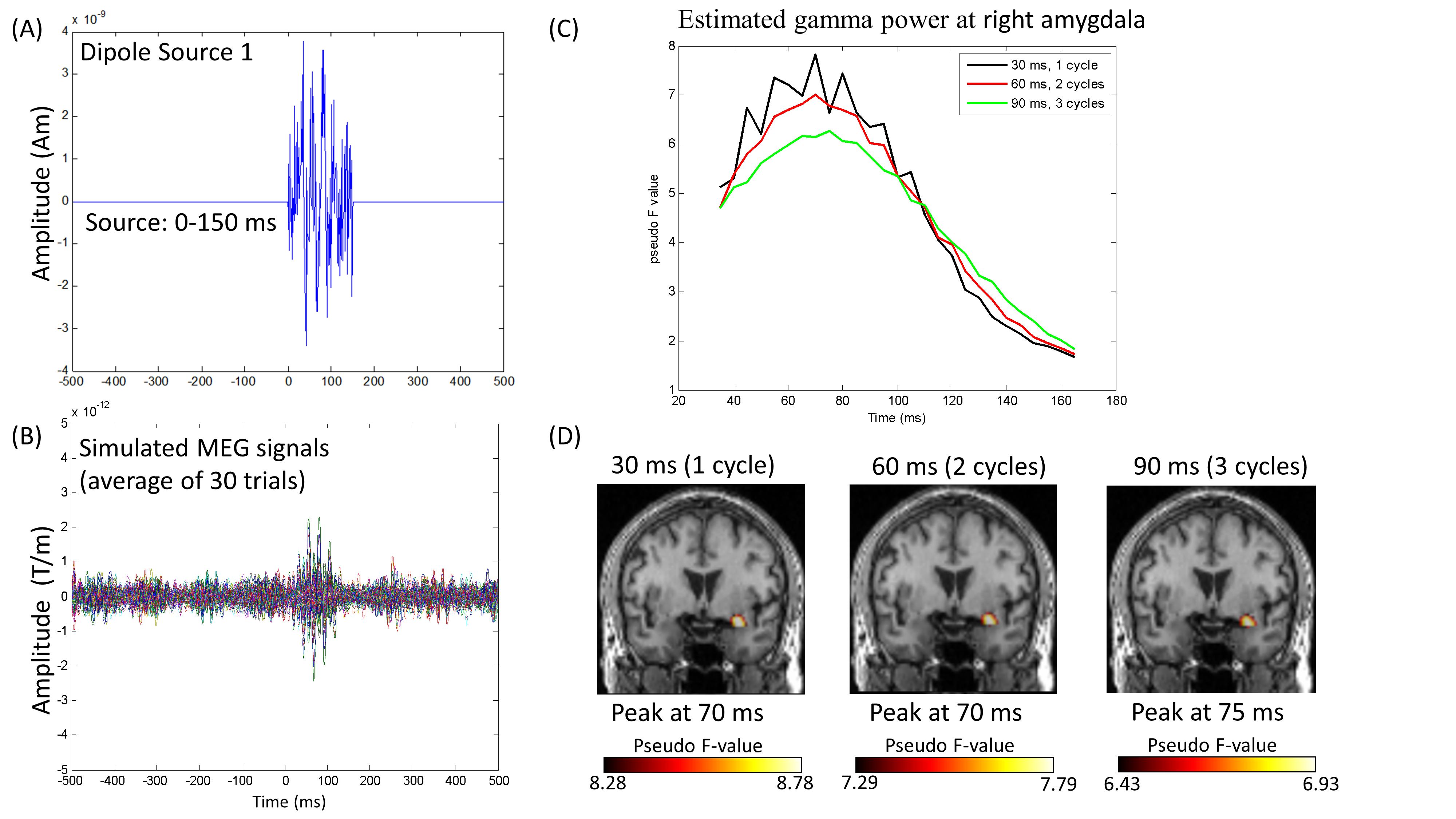

Supplement: S1 Fig — This figure illustrates (A) the temporal profile of the Dipole Source 1, (B) its corresponding simulated MEG sensor signals, (C) the temporal dynamics and (D) tomographic maps of the pseudo F-statistic values calculated by using the proposed method with different sizes of sliding window (30 ms, 60 ms, 90 ms). The simulated MEG data were originated from background activity and one dipole source located at the right amygdala (x = 30, y = −2, z = −26mm, MNI coordinates) with temporal profile of gamma-band sinusoidal waves added by random noises. The structural MRI data and MEG sensor configuration here were adopted from one subject in our facial processing experiment. (TIF) [file pone.0115677.s001.tif]

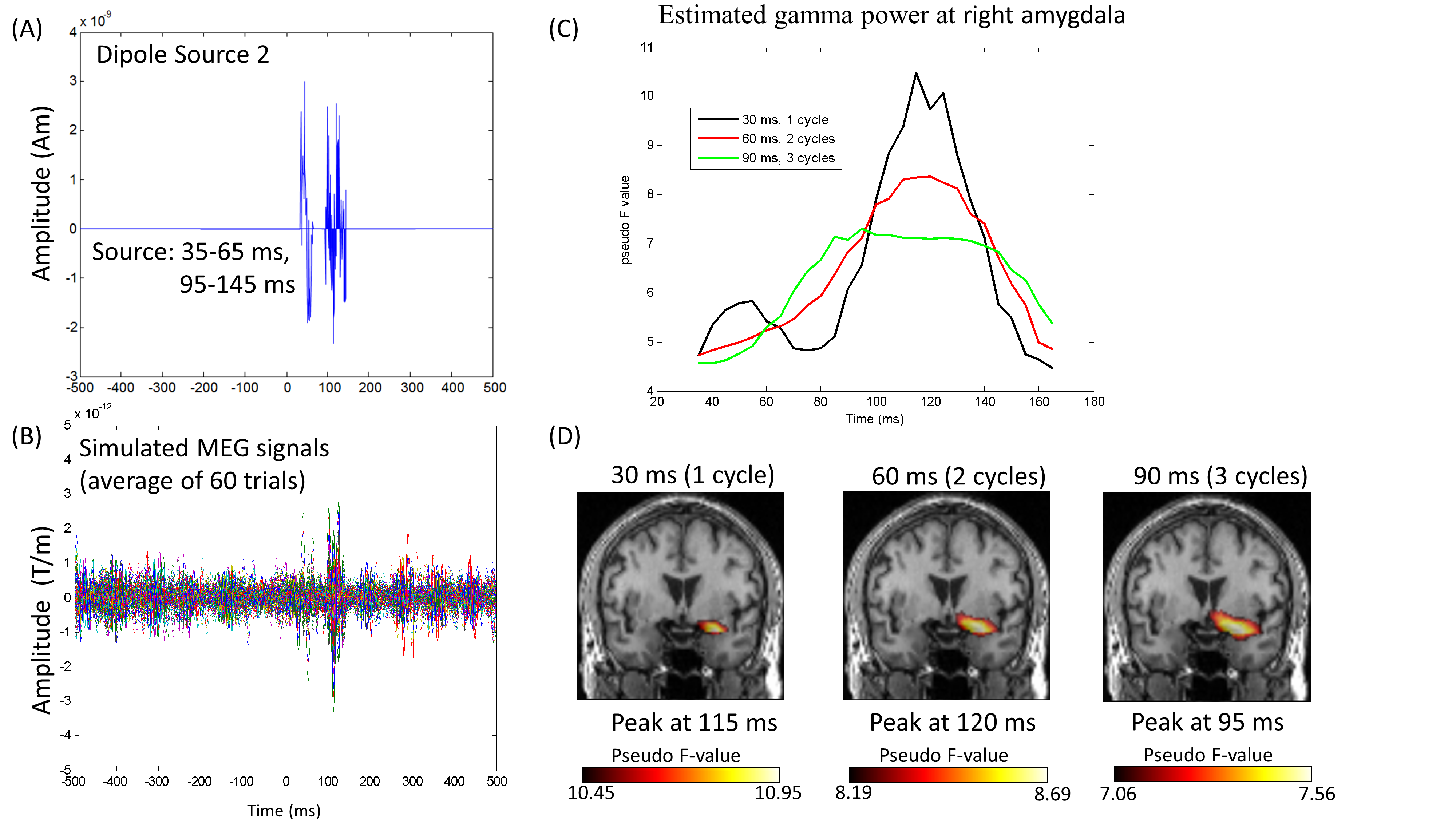

Supplement: S2 Fig — This figure illustrates (A) the temporal profile of the Dipole Source 2, (B) its corresponding simulated MEG sensor signals, (C) the temporal dynamics and (D) tomographic maps of the pseudo F-statistic values calculated by using the proposed method with different sizes of sliding window (30 ms, 60 ms, 90 ms). The simulated MEG data were originated from background activity and one dipole source located at the right amygdala (x = 30, y = −2, z = −26mm, MNI coordinates) with temporal profile of gamma-band sinusoidal waves added by random noises. The structural MRI data and MEG sensor configuration here were adopted from one subject in our facial processing experiment. (TIF) [file pone.0115677.s002.tif]

# A Angry facial expression

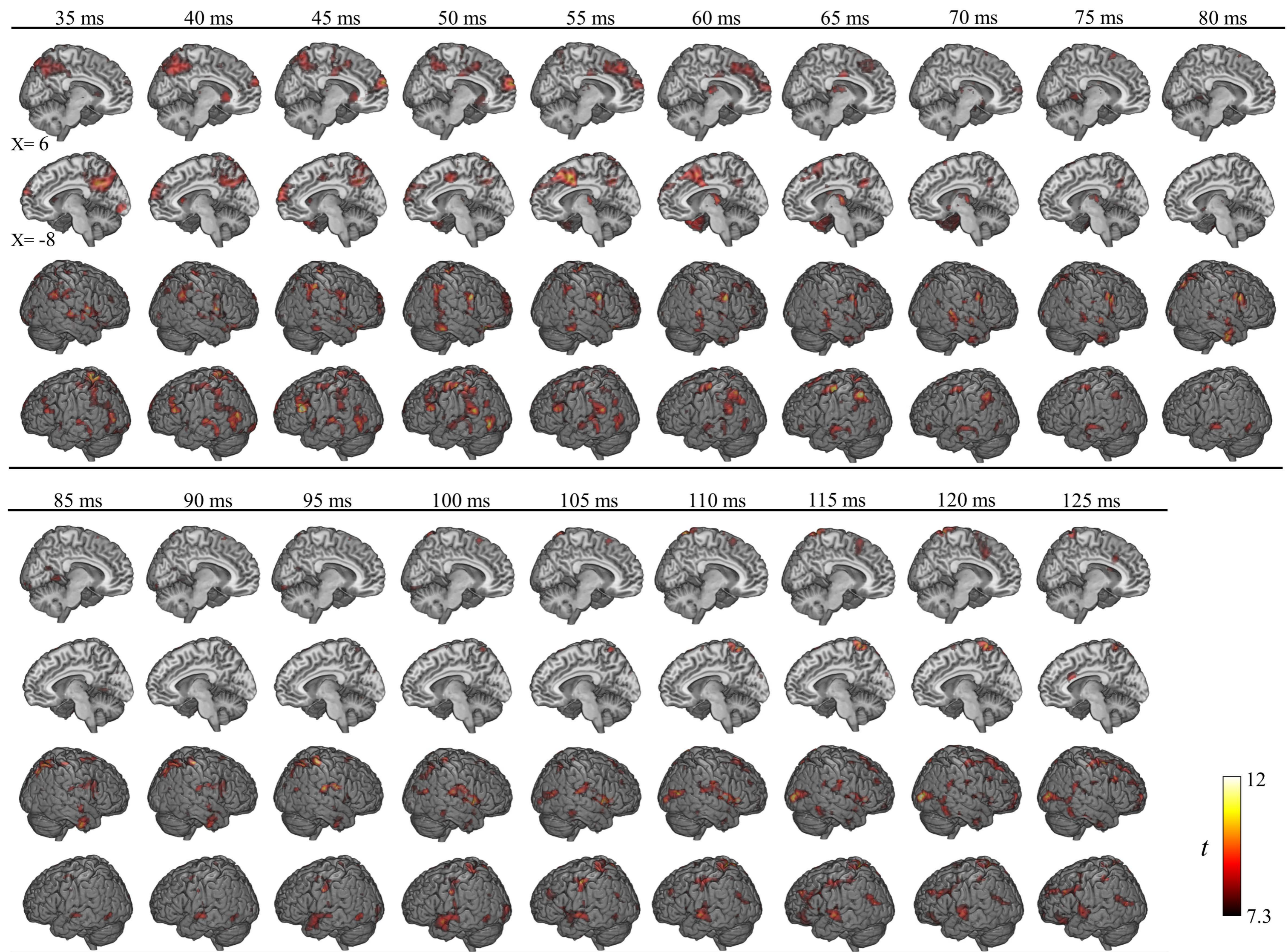

B Happy facial expression

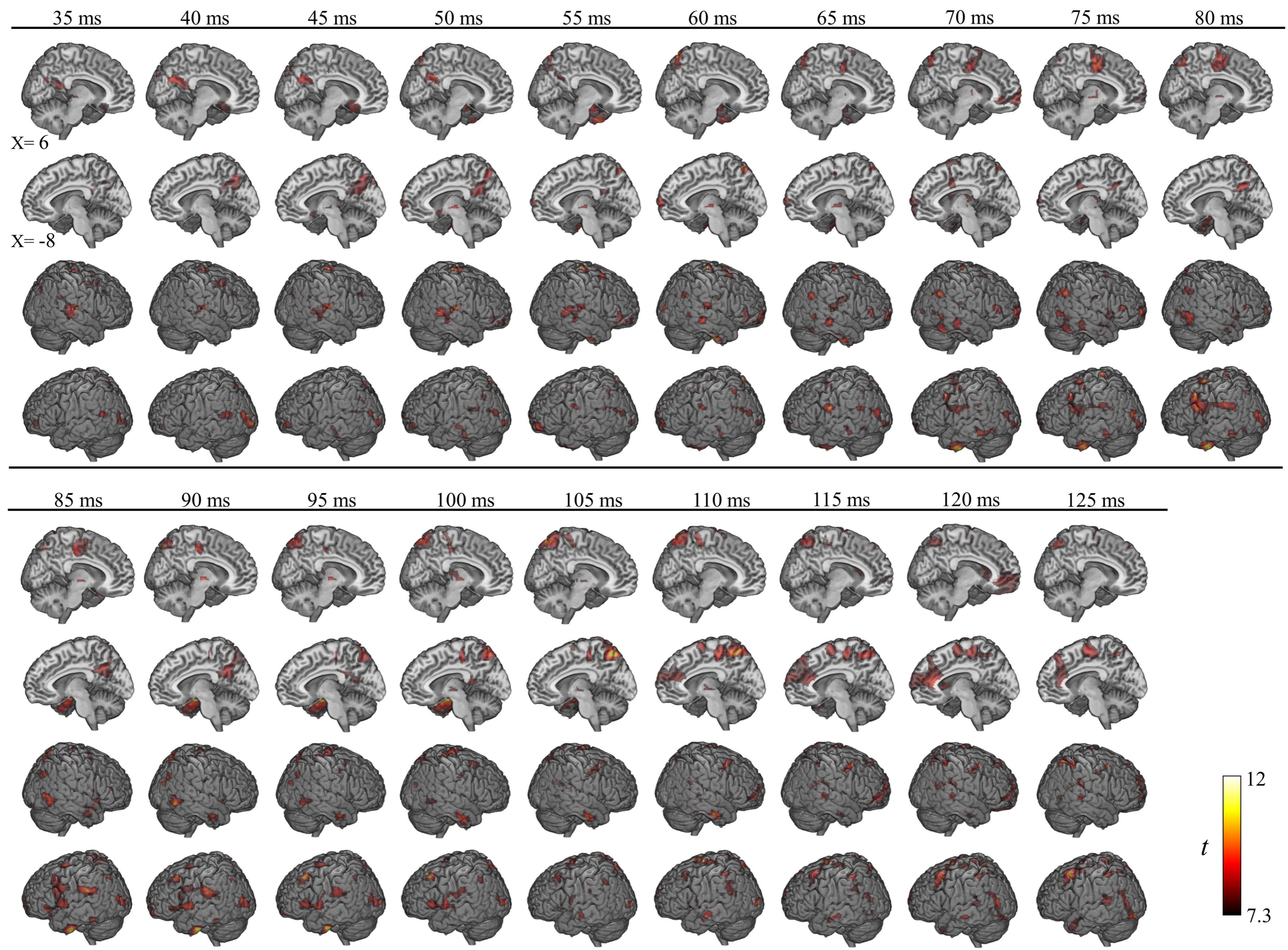

C Neutral facial expression

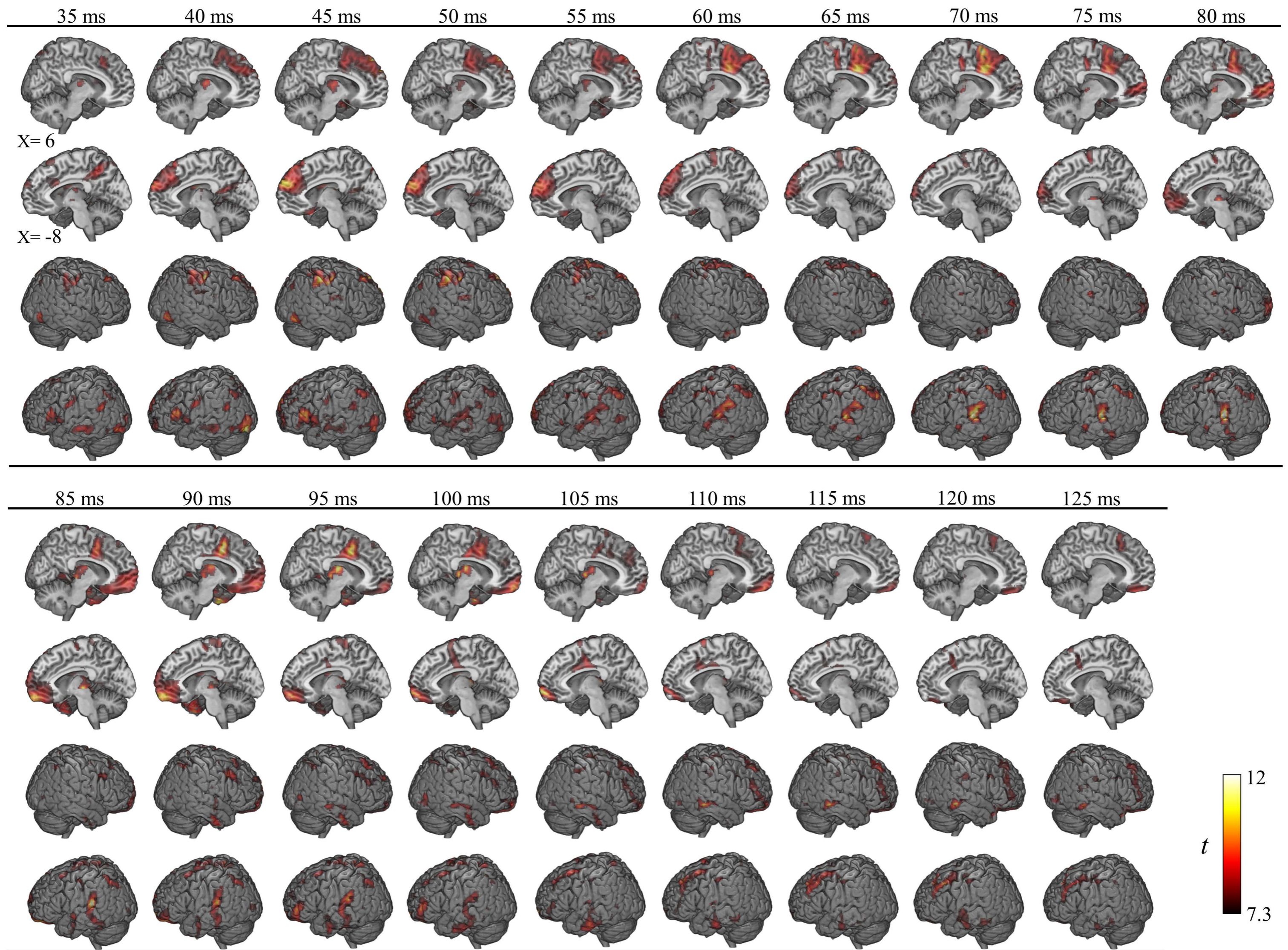

Supplement: S3 Fig — in response to the angry (A), happy (B), and neutral (C) faces during 35–125 ms after stimulus onset (extended cluster ≥ 10 voxels and t-value ≥ 7.3). (PDF) [file pone.0115677.s003.pdf]
